# Serum Gamma-Glutamyl Transpeptidase-to-Platelet Ratio as a Noninvasive Marker of Liver Fibrosis in Chronic Hepatitis B

**DOI:** 10.7759/cureus.33744

**Published:** 2023-01-13

**Authors:** Subham Purkayastha, Ashish K Jha, Ravikant Kumar, Vishwa Mohan Dayal, Sanjeev K Jha

**Affiliations:** 1 Gastroenterology, Indira Gandhi Institute of Medical Sciences, Patna, IND; 2 Gastroenterology and Hepatology, Indira Gandhi Institute of Medical Sciences, Patna, IND

**Keywords:** gamma-glutamyl transpeptidase (ggt)-to-platelet ratio, liver biopsy, transient elastography, fib-4 index, aspartate aminotransferase-to-platelet ratio index, liver fibrosis, chronic hepatitis b

## Abstract

Background

The gamma-glutamyl transpeptidase (GGT)-to-platelet ratio (GPR) is identified as a new model for the assessment of liver fibrosis in patients with chronic hepatitis B (CHB). We aimed to determine the diagnostic performance of GPR for the prediction of liver fibrosis in patients with CHB.

Methods

In an observational cohort study, patients with CHB were enrolled. The diagnostic performance of GPR was compared with transient elastography (TE), aspartate aminotransferase-to-platelet ratio index (APRI), and fibrosis-4 (FIB-4) scores for the prediction of liver fibrosis using liver histology as a gold standard.

Results

Forty-eight patients with CHB with a mean age of 33.42 ± 15.72 years were enrolled. Liver histology showed meta-analysis of histological data in viral hepatitis (METAVIR) stage F0, F1, F2, F3, and F4 fibrosis in 11, 12, 11, seven, and seven patients, respectively. The Spearman correlation of METAVIR fibrosis stage with APRI, FIB-4, GPR, and TE were 0.354, 0.402, 0.551, and 0.726, respectively (P value < 0.05). TE had the highest sensitivity, specificity, positive predictive value (PPV), and negative predictive value (NPV) (80%, 83%, 83%, and 79%, respectively), followed by GPR (76%, 65%, 70%, and 71%, respectively) for predicting significant fibrosis (≥F2). However, TE had comparable sensitivity, specificity, PPV, and NPV with GPR (86%, 82%, 42%, and 93%, and 86%, 71%, 42%, and 92%, respectively) for predicting extensive fibrosis (≥F3).

Conclusion

The performance of GPR is comparable to TE in predicting significant and extensive liver fibrosis. GPR may be an acceptable, low-cost alternative for predicting compensated advanced chronic liver disease (cACLD) (F3-F4) in CHB patients.

## Introduction

Approximately 240 million people are suffering from chronic hepatitis B virus (HBV) infection [1]. About 15%-40% of patients with chronic hepatitis B (CHB) progress to cirrhosis and end-stage liver disease [2]. Liver biopsy is still considered the gold standard for the assessment of the degree of hepatic inflammation and fibrosis [3]. However, liver biopsy has its inherent limitations such as invasiveness, sampling variability, cost, and complications. Furthermore, a single biopsy is not sufficient to measure the dynamic nature of liver fibrosis. The acceptability of repeated biopsy is very poor. Therefore, accurate, noninvasive, cost-effective, repeatable, and easily available alternative methods for identifying fibrosis in patients with CHB are warranted.

Recently, new noninvasive tests have been used to assess liver fibrosis. The most widely used noninvasive test is transient elastography (TE). It has shown good performance in assessing significant fibrosis (≥F2; area under the receiver operating characteristic (AUROC) curve: 0.65-0.97) and cirrhosis (F4; AUROC: 0.8-0.97) in patients with CHB. However, it is relatively less accurate in diagnosing significant fibrosis (≥F2) and more accurate in excluding extensive fibrosis (≥F3) or cirrhosis (F4). The usefulness of TE in patients with deep jaundice, high transaminasemia, obesity, and/or ascites is limited. The optimal diagnostic cutoff of TE for the stage of liver fibrosis in CHB is variable. In addition, TE is more expensive than other noninvasive markers [4].

Serum markers are alternative noninvasive models for assessing the degree of liver fibrosis [5]. Single markers are not specific enough to stage liver fibrosis. Thus, a combination of serum markers is used to obtain sufficient diagnostic accuracy. The majority of noninvasive serum markers for assessing liver fibrosis are validated in chronic hepatitis C. These tests include fibrosis-4 (FIB-4), FibroTest, aspartate transaminase-to-platelet ratio index (APRI), European Liver Fibrosis score, and Hepascore. However, a few noninvasive tests have been evaluated in assessing HBV-related liver fibrosis. FIB-4 and APRI are the most widely used and validated noninvasive tests for the prediction of HBV-related liver fibrosis. Studies have revealed that FIB-4 has optimal accuracy in identifying cirrhosis (F4) but is suboptimal in excluding significant fibrosis (≥F2). APRI has only moderate sensitivity and accuracy for assessing HBV-related fibrosis [5]. Therefore, better predicting noninvasive markers for patients with CHB with normal or mild transaminasemia are warranted. Recently, Lemoine et al. identified a new model for the assessment of liver fibrosis in patients with CHB: the gamma-glutamyl transpeptidase (GGT)-to-platelet ratio (GPR) [6]. The study showed that GPR is more accurate than APRI and FIB-4 in West Africa, but not superior to APRI and FIB-4 in France. There is a scarcity of data about the diagnostic value of GPR for liver fibrosis and cirrhosis in CHB patients. Most studies are conducted in China, and the results are variable. Studies from other Asian countries are scarcely available. The aim of the present study was to determine the diagnostic performance of GPR in patients with CHB in India.

## Materials and methods

This study was a prospective observational cohort study conducted at a tertiary care center. The study was approved by the Institutional Ethics Committee of Indira Gandhi Institute of Medical Sciences (IRB approval number: 432). Patients with CHB presenting to the Department of Gastroenterology were included in the study after obtaining written informed consent. The inclusion criteria were patients with hepatitis B surface antigen (HBsAg) positivity for >6 months, treatment-naive patients, and age ≥ 18 years. The exclusion criteria were hepatitis C virus (HCV), hepatitis D (HDV) or human immunodeficiency virus (HIV) confection, prior or concurrent antiviral therapy, hepatocellular carcinoma (HCC), concomitant tuberculosis, patients with non-HBV-related chronic liver disease including nonalcoholic steatohepatitis (NASH), sepsis, grossly elevated transaminase levels (>10 times upper limit of normal (ULN)), significant alcohol consumption (>20 g/day), heart failure, and pregnancy.

Detailed clinical history and physical examination of patients were done. All patients were tested for complete blood count, liver function test, prothrombin time, blood sugar, renal function test, alpha-fetoprotein, HBsAg, hepatitis B envelop (HBe) antigen, anti-HBe antibody, anti-hepatitis B core (HBc) antibody, HBV deoxyribonucleic acid (DNA) by quantitative polymerase chain reaction (PCR), HIV, and anti-HCV antibody. Appropriate tests for autoimmune liver disease, Wilson disease, and hemochromatosis were performed. Chest radiograph and abdominal ultrasonography (US) were also performed, if not done earlier. Computed tomography scan of the whole abdomen and endoscopy were done whenever required.

Platelet count was estimated using the Sysmex XN-550 automated complete blood count analyzer (Sysmex Corporation, Kobe, Japan) (normal reference values: 150-410 × 10^9^/L). Manual counting was performed using a Neubauer counting chamber if the automated analyzer gave an asterisk flag or the platelet count was less than 1 lac/mm^3^. GGT was measured using the Cobas Integra 400 analyzer (Roche Diagnostics, Mannheim, Germany) (normal reference values for females: 6-42 IU/L, normal reference values for males: 10-71 IU/L). GPR was calculated as ((GGT (IU/L) / ULN) / platelet count (10^9^/L)) × 100, APRI was calculated as ((aspartate aminotransferase (AST) (IU/L) / ULN) / platelet count (10^9^/L)) × 100, and FIB-4 was calculated as (age (years) × AST (IU/L)) / (platelet count (10^9^/L) × √ (alanine transaminase (ALT) (IU/L))). TE was done using Fibroscan-402 (Echosens, Paris, France) according to the manufacturer’s protocol in the fasting state. The value of liver stiffness measurement (LSM) was expressed in kilo pascal (kPa) as the median of 10 successful acquisitions. US-guided percutaneous liver biopsy was done in patients with any of the following features in the absence of contraindications: AST or ALT ≥ 40 IU/L, LSM ≥ 6.5 kPa, a family history of cirrhosis or HCC in a first-degree relative, and HBV DNA ≥ 2,000 IU/mL. Liver biopsy was performed using 18-G disposable needles of cutting type (BARD MAXCORE Disposable Core Biopsy Instrument, Bard Biopsy Systems, Covington, USA). Tissue was fixed in 10% formalin and stained with hematoxylin and eosin and Masson’s trichrome. All liver biopsy samples were evaluated by a pathologist who was kept blind to the clinical details, laboratory data, and imaging findings. Patients with meta-analysis of histological data in viral hepatitis (METAVIR) stage F0, F1, F2, F3, and F4 were classified as having nonsignificant fibrosis (F0-F1), significant fibrosis (≥F2), extensive fibrosis (≥F3), and cirrhosis (F4). A liver biopsy showing severe fibrosis (F3) or established cirrhosis (F4) confirms the diagnosis of compensated advanced chronic liver disease (cACLD) as per the Baveno VI consensus report [7]. The diagnostic performance of GPR was compared with that of TE, APRI, and FIB-4 tests for the prediction of the degree of liver fibrosis using liver histology as a gold standard. The diagnostic performance of GPR was evaluated for the diagnosis of significant liver fibrosis and extensive liver fibrosis/cACLD.

Statistical analysis

All continuous variables were expressed as mean ± standard deviation (SD) and those with noncontinuous distribution as median. A chi-squared test was used to assess the association between categorical variables. Student’s t-test was performed to compare the means between the two groups. The relationship between the noninvasive biomarkers and liver histopathology was determined using Spearman’s rank correlation coefficient analysis. The diagnostic performance of all noninvasive markers was assessed using receiver operating characteristic (ROC) curves using histology as a reference. DeLong’s test was done to compare the area under the receiver operating characteristic curve (AUROC) to find any difference in the performance of the noninvasive tests. A P value < 0.05 was considered significant.

## Results

Out of 99 patients assessed for eligibility for the study, 48 were finally enrolled. Fifty-one patients were excluded because of refusal for liver biopsy (n = 23), grossly raised transaminase levels (n = 12), prior treatment (n = 7), HCC (n = 4), significant alcohol intake (n = 3), and concomitant HIV or HCV (n = 1 each). Hepatitis B envelope antigen (HBeAg) was absent in 78% (32/41) of patients having no cirrhosis, while only 28.57% (2/7) of patients with cirrhosis (F4) had absent HBeAg (P = 0.017). The baseline characteristics of the patients are shown in Table 1.

**Table 1:** Baseline characteristics of the patient population SD: standard deviation, ALT: alanine transaminase, AST: aspartate transaminase, ALP: alkaline phosphatase, GGT: gamma-glutamyl transpeptidase, HBeAg: hepatitis B envelope antigen, HBV DNA: hepatitis B virus deoxyribonucleic acid

Parameter	Mean ± SD
Total cases (n)	48
Age (years)	33.42 ± 15. 72
Sex (%)	85.42 (male)
HBeAg negative (%)	70.83
Hemoglobin (g/dL)	12.86 ± 2.05
Platelet count (×10^9^/L)	175.81 ± 64.83
Total bilirubin (mg/dL)	0.82 ± 0.41
ALT (IU/L)	60.43 ± 56.81
AST (IU/L)	49.89 ± 36.36
ALP (IU/L)	153.21 ± 61.72
GGT (IU/L)	26.91 ± 14.49
Serum albumin (g/dL)	4.2 ± 0.5
HBV DNA (IU/mL)	4745278.44 ± 19530772.04

METAVIR stage F0, F1, F2, F3, and F4 were observed in 11 (22.92%), 12 (25%), 11 (22.92%), seven (14.58%), and seven (14.58%) cases, respectively. Platelet count (200.65 versus 152.96 × 10^9^/L) and GGT (21.89 versus 31.54 IU/L) were significantly different between the F0-F1 and ≥F2 group (P = 0.01 and 0.019, respectively). Thirty-four patients had ≤F2 fibrosis, and 14 patients had ≥F3 fibrosis. Platelet count, GGT, and serum albumin were significantly different between the ≤F2 and ≥F3 group (195.24 ± 60.05 versus 128.64 ± 51.56, P = 0.001; 23.23 ± 11.78 versus 35.86 ± 16.88, P = 0.020; 4.33 ± 0.37 versus 3.87 ± 0.64, P = 0.023, respectively) (Table 2).

**Table 2:** Comparison of baseline characteristics (mean ± SD) SD: standard deviation, ALT: alanine transaminase, AST: aspartate transferase, ALP: alkaline phosphatase, GGT: gamma-glutamyl transpeptidase, HBeAg: hepatitis B envelope antigen, HBV DNA: hepatitis B virus deoxyribonucleic acid

Parameters	Nonsignificant (F0-F1) (n = 23)	Significant (≥F2) (n = 25)	P value	Non-extensive (≤F2) (n = 34)	Extensive (≥F3) (n = 14)	P value
Age (years)	28.96 ± 11.91	37.52 ± 17.82	0.055	31.11 ± 13.73	39.0 ± 19.18	0.179
Male sex (%)	78.26	92	0.237	82.35	92.86	0.656
HBeAg negative (%)	78.26	64	0.278	79.41	50	0.078
Hemoglobin (g/dL)	12.87 ± 1.61	12.85 ± 2.42	0.976	13.09 ± 1.70	12.31 ± 2.71	0.338
Platelet count (10^9^/L)	200.65 ± 62.24	152.96 ± 59.53	0.010	195.24 ± 60.05	128.64 ± 51.56	0.001
Bilirubin (mg/dL)	0.78 ± 0.37	0.87 ± 0.45	0.457	0.79 ± 0.42	0.92 ± 0.38	0.309
ALT (IU/L)	59.48 ± 73.38	61.30 ± 37.18	0.916	59.09 ± 62.96	63.67 ± 39.87	0.764
AST (IU/L)	43.35 ± 37.83	55.90 ± 34.61	0.238	45.16 ± 32.85	61.36 ± 42.90	0.220
ALP (IU/L)	136.04 ± 60.71	169 ± 59.49	0.064	145.26 ± 57.61	172.50 ± 69.14	0.208
GGT (IU/L)	21.89 ± 13.19	31.54 ± 14.33	0.019	23.23 ± 11.78	35.86 ± 16.88	0.020
Serum albumin (g/dL)	4.31 ± 0.41	4.10 ± 0.57	0.152	4.33 ± 0.37	3.87 ± 0.64	0.023
HBV DNA (IU/mL)	4942207 ± 19557891	4564104 ± 19907330	0.947	6389817 ± 23091227	751398 ± 1149378	0.165

The sensitivity, specificity, positive predictive value (PPV), and negative predictive value (NPV) of GPR, TE, APRI, and FIB-4 for the prediction of fibrosis in patients with CHB are shown in Table 3. TE had the highest sensitivity, specificity, PPV, and NPV (80%, 83%, 83%, and 79%, respectively), followed by GPR (76%, 65%, 70%, and 71%, respectively) for the prediction of significant fibrosis (≥F2). However, TE had comparable sensitivity, specificity, PPV, and NPV with GPR (86%, 82%, 42%, and 93%, and 86%, 71%, 42%, and 92%, respectively) for the prediction of extensive fibrosis (≥F3). Spearman’s correlation of the METAVIR fibrosis stage with APRI, FIB-4, GPR, and TE were 0.354, 0.402, 0.551, and 0.726, respectively, with P value < 0.01 for all four tests.

**Table 3:** Sensitivity, specificity, positive predictive value, and negative predictive value of tests GPR: gamma-glutamyl transpeptidase-to-platelet ratio, APRI: aspartate aminotransferase-to-platelet ratio index, FIB-4: fibrosis-4 index, TE: transient elastography, Sn: sensitivity, Sp: specificity, PPV: positive predictive value, NPV: negative predictive value

Fibrosis stages	Significant fibrosis (≥F2)					Extensive fibrosis (≥F3)				
Test	Cutoff value	Sn (%)	Sp (%)	PPV (%)	NPV (%)	Cutoff value	Sn (%)	Sp (%)	PPV (%)	NPV (%)
APRI	0.46	68	57	63	62	0.54	71	59	42	83
FIB-4	0.78	72	52	62	63	0.82	71	50	42	81
GPR	0.28	76	65	70	71	0.32	86	71	42	92
TE	7.7	80	83	83	79	8.8	86	82	42	93

**Figure 1:**
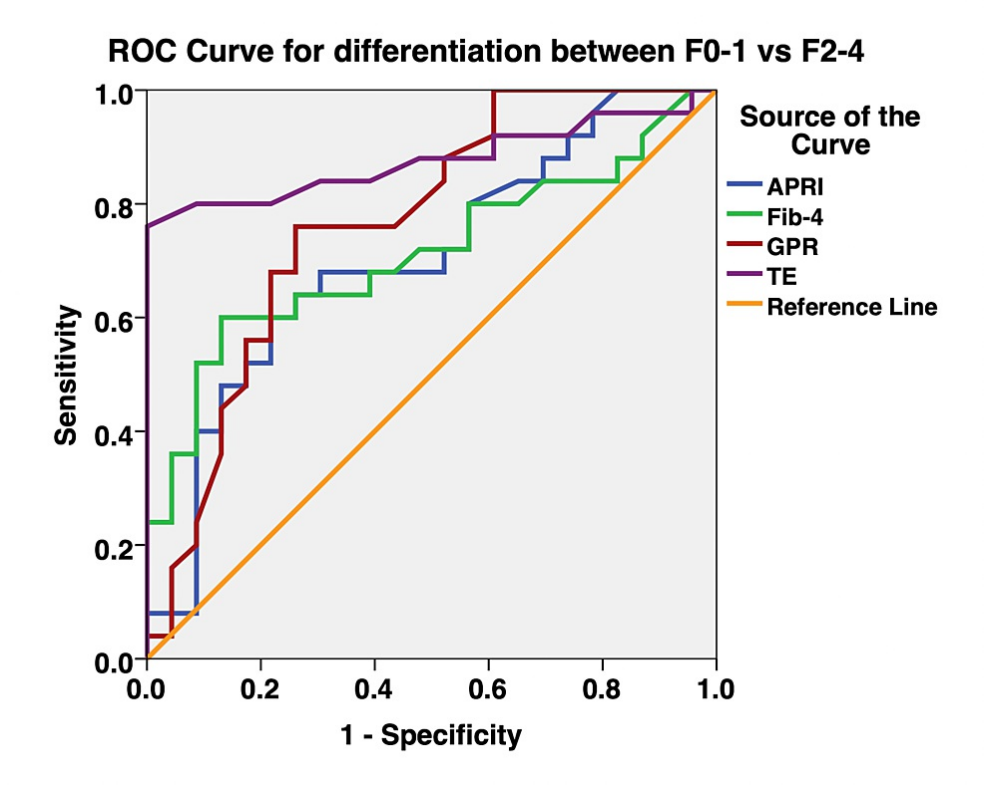
Receiver operating characteristic curves for the differentiation of liver fibrosis stages (≥F2) ROC: receiver operating characteristic, APRI: aspartate aminotransferase-to-platelet ratio index, FIB-4: fibrosis-4 index, GPR: gamma-glutamyl transpeptidase-to-platelet ratio, TE: transient elastography

**Figure 2:**
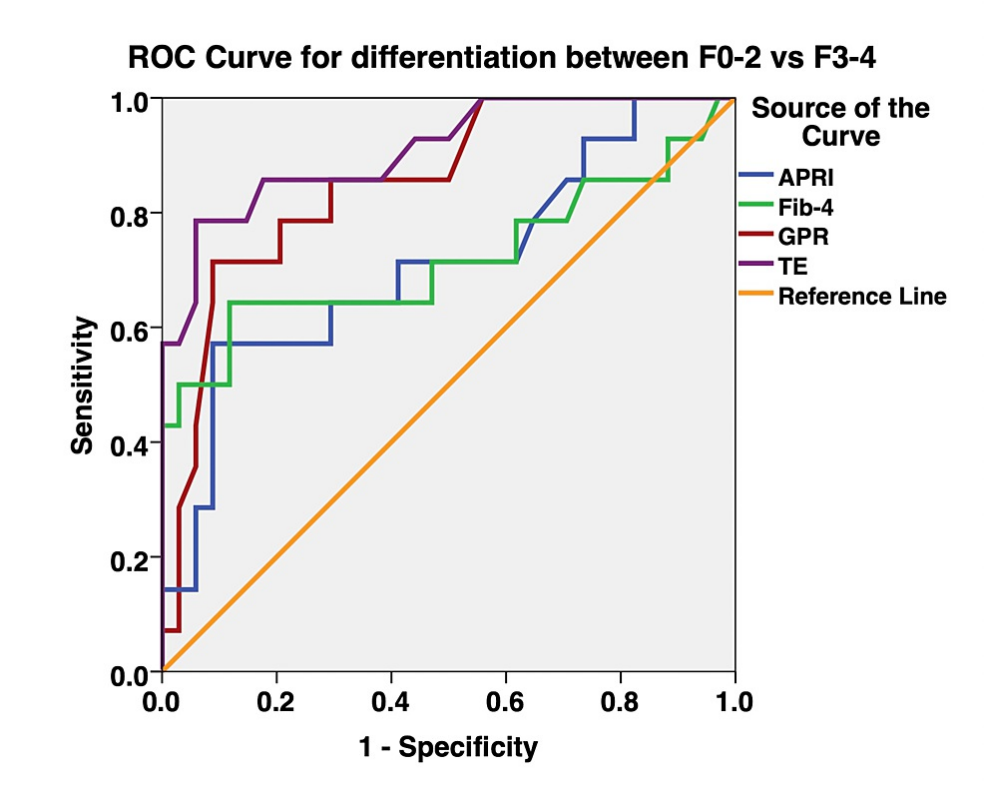
Receiver operating characteristic curves for the differentiation of liver fibrosis stages (≥F3) ROC: receiver operating characteristic, APRI: aspartate aminotransferase-to-platelet ratio index, FIB-4: fibrosis-4 index, GPR: gamma-glutamyl transpeptidase-to-platelet ratio, TE: transient elastography

The diagnostic performance of GPR, APRI, FIB-4, and TE was assessed using the AUROC curve. The AUROC for the prediction of fibrosis stages ≥F2 (Figure 1) and ≥F3 (Figure 2) were greatest for TE, followed by GPR. In comparison, TE had greater AUROC than GPR for the prediction of significant and extensive liver fibrosis, but there were no statistically significant differences observed. The AUROC for the prediction of significant and extensive liver fibrosis were significantly greater for TE compared to APRI (Table 4 and Table 5). The AUROC for the prediction of significant and extensive liver fibrosis was significantly greater for TE compared to FIB-4 (Table 4 and Table 5). The performance of GPR was better than that of APRI and FIB-4 in terms of sensitivity, specificity, PPV, and NPV (Table 3). The AUROC of GPR for the prediction of significant and extensive liver fibrosis was better than that of APRI and FIB-4, but there were no statistically significant differences observed between GPR and APRI (P = 0.1241), and GPR and FIB-4 (P = 0.3761) (Figure 1 and Figure 2, Table 4 and Table 5).

**Table 4:** AUROC (95% CI) for fibrosis stages AUROC: area under the receiver operating characteristic curves, GPR: gamma-glutamyl transpeptidase-to-platelet ratio, APRI: aspartate aminotransferase-to-platelet ratio index, FIB-4: fibrosis-4 index, CI: confidence interval, vs: versus

Test	Nonsignificant (F0-F1) vs significant (≥F2) fibrosis			Non-extensive (F0-F2) vs extensive (≥F3) fibrosis		
	AUROC	CI	P value	AUROC	CI	P value
APRI	0.703	0.554-0.853	0.016	0.711	0.539-0.883	0.023
FIB-4	0.713	0.564-0.862	0.011	0.721	0.528-0.913	0.017
GPR	0.768	0.631-0.905	0.001	0.853	0.737-0.969	0.0001
TE	0.877	0.769-0.986	<0.0001	0.905	0.809-1.000	0.0001

**Table 5:** Comparison of AUROC for the prediction of fibrosis AUROC: area under the receiver operating characteristic curves, GPR: gamma-glutamyl transpeptidase-to-platelet ratio, APRI: aspartate aminotransferase-to-platelet ratio index, FIB-4: fibrosis-4 index, TE: transient elastography, vs: versus

Compared tests	Significant fibrosis (≥F2) (P value)	Extensive fibrosis (≥F3) (P value)
GPR vs APRI	0.3926	0.0843
GPR vs FIB-4	0.4300	0.0709
GPR vs TE	0.1780	0.3581
TE vs APRI	0.0320	0.0343
TE vs FIB-4	0.0382	0.0280
APRI vs FIB-4	0.8909	0.8986

## Discussion

The acceptability of liver biopsy is very poor due to its invasive nature and potential complications. TE is a widely used test for determining the degree of fibrosis in chronic liver diseases, but it is limited by availability and cost [8,9]. The current study shows that TE is the best noninvasive predictor of significant fibrosis and extensive fibrosis/cACLD in patients with CHB. The diagnostic performance of GPR is nearly comparable to that of TE for staging liver fibrosis. GPR had better predictability compared to APRI and FIB-4.

In a multicenter study, Lemoine et al. reported good performance of GPR in training as well as validation cohorts [6]. In The Gambia, the AUROC of GPR was significantly higher than that of APRI and FIB-4 in predicting ≥F2, ≥F3, and F 4. In Senegal, the AUROC of GPR was significantly better than FIB-4 and APRI for ≥F2 (0.73; 95% confidence interval (CI): 0.59-0.86) and better than FIB-4 and TE for ≥F3 (0.93; 95% CI: 0.87-0.99). However, the AUROC of GPR to diagnose ≥F2 (0.72; 95% CI: 0.59-0.85) and F4 (0.87; 95% CI: 0.76-0.98) was equivalent to that of APRI and FIB-4 in a French cohort. Ren et al. showed that the AUROC of GPR for fibrosis was significantly higher than that of APRI (≥F1: 0.77 versus (vs) 0.70, P = 0.03; ≥F2: 0.70 vs 0.63, P = 0.02; ≥F3: 0.71 vs 0.64, P = 0.02) but comparable to that of FIB-4 (≥F1: 0.77 vs 0.75, P = 0.14; ≥F2: 0.70 vs 0.70, P = 0.22; ≥F3: 0.71 vs 0.68, P = 0.13; F4: 0.64 vs 0.67, P = 0.24) [10]. Liu et al. showed that regardless of HBeAg status, GPR had the best performance in predicting liver fibrosis stage when compared with APRI and FIB-4. There was no significant difference in the AUROC of GPR, APRI, and FIB-4 in HBeAg-positive patients for predicting significant fibrosis, but the AUROC for GPR was larger than APRI for predicting extensive fibrosis and cirrhosis (P = 0.0001 and P < 0.0001). In HBeAg-negative patients, the AUROCs of GPR in predicting significant fibrosis and cirrhosis were larger than those of FIB-4 (P = 0.0006 and P = 0.0041). The AUROC of GPR in predicting extensive fibrosis was larger than that of APRI and FIB-4 (P = 0.0320 and P = 0.0018) [11]. In a study by Li et al., the AUROC of GPR, APRI, and FIB-4 were compared for the prediction of liver fibrosis stage in HBeAg-positive patients with HBV DNA ≥ 5 log10 copies/mL and ALT ≤ 2 ULN. GPR had the best performance in predicting the different stages of liver fibrosis and cirrhosis [12].

The findings of studies performed to determine the diagnostic performance of GPR are summarized in Table 6 [6,10-21]. Differences in study findings can be explained by the adoption of different staging systems and the sample size in some of the study cohorts. Most of the published studies where the METAVIR staging system was adopted showed significantly better or comparable performance of GPR compared to APRI and FIB-4 for predicting HBV-related fibrosis [6,10-15,19,21]. In the current study, GPR performed better than APRI and FIB-4 in terms of AUROC, but there was no statistically significant difference. Other authors have also reported nearly comparable results [6,10-15,19,21]. In these reports, fibrosis were staged with the METAVIR staging system. Studies where the Scheuer scoring system was adopted as the pathological standard of liver fibrosis showed mostly comparable performance of GPR for the prediction of HBV-related fibrosis compared to APRI and/or FIB-4 [16-18]. However, Li et al. reported that APRI was better than GPR for predicting extensive fibrosis and cirrhosis [18].

**Table 6:** Review of literature CHB: chronic hepatitis B virus, HBeAg: hepatitis B envelope antigen, GPR: gamma-glutamyl transpeptidase-to-platelet ratio, APRI: aspartate aminotransferase-to-platelet ratio index, FIB-4: fibrosis-4 index, TE: transient elastography, AUROC: area under the receiver operating characteristic curves, NAFLD: nonalcoholic fatty liver disease

References	Cases (n)	Country	Conclusion
Lemoine et al. [6]	CHB (135)	The Gambia	AUROC GPR > APRI and FIB-4 to predict ≥F2, ≥F3, and F4 (P < 0.05)
	CHB (63)	France	AUROC GPR = APRI and FIB-4 to predict ≥F2 and F4 (P > 0.05)
	CHB (80)	Senegal	AUROC GPR > APRI and FIB-4 to predict ≥F2 (P < 0.05); GPR > FIB-4 and TE to predict ≥F3 (P < 0.05)
Li et al. [12]	CHB (401)		HBeAg-positive AUROC GPR > APRI and FIB-4 to predict ≥F2, ≥F3, and F4 (P < 0.05)
Wang et al. [13]	CHB (312)	China	AUROC GPR > APRI and FIB-4 to predict ≥F2, ≥F3, and F4 (P < 0.05)
Ren et al. [10]	CHB (160)	China	AUROC GPR > APRI to predict ≥F2 and ≥F3 (P < 0.05); AUROC GPR = FIB-4 to predict ≥F2, ≥F3, and F4 (P > 0.05)
Hu et al. [14]	CHB (390)	China	AUROC GPR > APRI and FIB-4 to predict ≥F2, ≥F3, and F4 (P < 0.05)
Huang et al. [15]	CHB (256)	China	AUROC GPR = APRI = FIB-4 to predict ≥F2, ≥F3, and F4 (P > 0.05)
Liu et al. [11]	CHB (2,016)	China	HBeAg-positive AUROC GPR > APRI to predict ≥F3 and F4 (P < 0.05); HBeAg-negative AUROC GPR > APRI and FIB-4 to predict ≥F3 (P < 0.05); HBeAg-negative AUROC GPR > FIB-4 to predict ≥F2 (P < 0.05) and F4
Wu et al. [16]	CHB (323)	China	AUROC GPR = APRI = FIB-4 to predict ≥S2 (P > 0.05); AUROC GPR and APRI > FIB-4 to predict ≥S3 (P < 0.05)
Wang et al. [17]	CHB (370)	China	AUROC GPR = APRI = FIB-4 to predict ≥S2 and ≥S3 (P > 0.05)
Li et al. [18]	CHB (372)	China	AUROC GPR < APRI to predict ≥S2, ≥S3, and ≥S4 (P > 0.05); AUROC GPR = FIB-4 to predict ≥S2, ≥S3, and ≥S4 (P > 0.05)
Li et al. [19]	CHB-NAFLD (131)	China	AUROC GPR > APRI and FIB-4 to predict ≥F2 and ≥F3 (P < 0.05); AUROC GPR = APRI to predict F4 (P < 0.05)
Zhang et al. [20]	CHB-NAFLD (145)	China	AUROC GPR < TE to predict F4 (P < 0.05)
Khare et al. [21]	CHB (79)	India	AUROC GPR, APRI, and TE to predict Ishak > 3 (P > 0.05)

There is a wide variation in GPR cutoff levels for the prediction of different stages of fibrosis in study cohorts. Variations in the cutoff level were mainly explained by the use of different histopathological scoring systems (METAVIR, Scheuer fibrosis scoring system, and modified Ishak grading system). Different cutoff levels can also be explained by different study cohorts and the small sample size in some of the studies. In our study, the optimal cutoff value of GPR for the prediction of significant fibrosis was 0.28. The optimal cutoff values for GPR reported in other studies were 0.32 (Lemoine et al. [6]), 0.35 (Ren et al. [10]), 0.34 (Huang et al. [15]), 0.61 (Li et al. [19]), and 0.46 (Wang et al. [13]). The optimal cutoff value in this study also differs from a recent Indian study where the optimal cutoff for predicting significant fibrosis was 0.44. However, fibrosis staging was done according to the modified Ishak grading system. The number of patients in the significant fibrosis group was small (n = 15) [21].

The optimal cutoff values of GPR for predicting extensive fibrosis are also variable. The optimal cutoff value of GPR for predicting extensive fibrosis was 0.32 in the current study. Lemoine et al. reported an optimal cutoff value of 0.32 for GPR in both the training set in The Gambia and the validation sets in France and Senegal [6]. For the prediction of extensive fibrosis, Wang et al. reported a cutoff value of 0.56 [13], while Li et al. reported a cutoff value of 0.65 [19]. The optimal cutoff values of GPR for predicting extensive fibrosis, according to other studies, were 0.41 (Huang et al. [15]) and 0.38 (Ren et al. [10]).

A study by Lemoine et al. revealed significantly better performance of GPR compared with TE for the prediction of extensive fibrosis ≥F3 (P < 0.05) in patients with CHB [6]. However, another study by Zhang et al. showed significantly poorer performance of GPR compared to TE for the prediction of F4 (P < 0.05) in patients with CHB concurrent with nonalcoholic fatty liver disease (NAFLD) [20]. Our study showed nearly comparable performance of GPR and TE for the prediction of significant fibrosis and extensive fibrosis. However, the performance of TE for the prediction of significant fibrosis and extensive fibrosis was significantly better compared to APRI and FIB-4. The findings of studies performed to determine the diagnostic performance of GPR in CHB patients are summarized in Table 6 [6,10-21].

Limitations

This study, however, has its limitations. The indication for liver biopsy depended on the results of the other investigations (AST, ALT, and TE). Therefore, the current study cohort may not represent the general population with CHB. Histopathological examination of the liver biopsy specimen was performed by a single pathologist, and the opinion of a second pathologist for the interobserver agreement of the histological fibrosis stage was not sought. We excluded patients with significant alcohol consumption (>20 g/day). However, GGT can be potentiated by alcohol intake as low as 7-14 g/day. The ideal would be to take those abstinent over the last two months at least. Finally, this study is also limited by the small sample size.

## Conclusions

TE is the best noninvasive predictor of significant fibrosis and extensive fibrosis. The widespread use of TE is limited by higher costs and availability. GPR is a simple, easy-to-calculate, inexpensive model for the prediction of the degree of liver fibrosis in CHB patients. The performance of GPR is comparable to TE in predicting significant and extensive liver fibrosis. GPR has comparatively better performance than APRI and FIB-4 for predicting liver fibrosis. GPR may be an acceptable, low-cost alternative for predicting cACLD (F3-F4) in CHB patients. Due to variability in the GPR cutoff level for the prediction of liver fibrosis, this test requires multicentric validation and finding of the appropriate cutoff level.
